# Glial Fibrillary Acidic Protein Is Not an Early Marker of Injury in Perinatal Asphyxia and Hypoxic–Ischemic Encephalopathy

**DOI:** 10.3389/fneur.2015.00264

**Published:** 2015-12-21

**Authors:** Ann-Marie Looney, Caroline Ahearne, Geraldine B. Boylan, Deirdre M. Murray

**Affiliations:** ^1^Neonatal Brain Research Group, Department of Paediatrics and Child Health, Irish Centre for Fetal and Neonatal Translational Research, Cork University Maternity Hospital, Cork, Ireland

**Keywords:** GFAP, hypoxic–ischemic encephalopathy, early diagnosis, biomaker, umbilical cord blood, therapeutic interventions

## Abstract

Brain-specific glial fibrillary acidic protein (GFAP) has been suggested as a potential biomarker for hypoxic ischemic encephalopathy (HIE) in newborns (1, 2). Previous studies have shown increased levels in post-natal blood samples. However, its ability to guide therapeutic intervention in HIE is unknown. Therapeutic hypothermia for HIE must be initiated within 6 h of birth, therefore a clinically useful marker of injury would have to be available immediately following delivery. The goal of our study was to examine the ability of GFAP to predict grade of encephalopathy and neurological outcome when measured in umbilical cord blood (UCB). Infants with suspected perinatal asphyxia (PA) and HIE were enrolled in a single, tertiary maternity hospital, where UCB was drawn, processed, and bio-banked at birth. Expression levels of GFAP were measured by ELISA. In total, 169 infants (83 controls, 56 PA, 30 HIE) were included in the study. GFAP levels were not increased in UCB of case infants (PA/HIE) when compared to healthy controls or when divided into specific grades of HIE. Additionally, no correlation was found between UCB levels of GFAP and outcome at 36 months.

## Introduction

Perinatal asphyxia (PA) occurs when there is a disruption of oxygen delivery or blood supply to the fetus around time of birth. PA, when severe, leads to hypoxic–ischemic encephalopathy (HIE) in the neonatal period. HIE remains one of the leading clinical challenges faced in the neonatal period and the greatest cause of acquired brain injury in term infants. To optimize outcomes in neonatal HIE, early and accurate prediction of the degree of encephalopathy is vital. Neuroprotective therapies must be commenced prior to the development of secondary brain injury, giving a narrow therapeutic window of <6 h after delivery (3). Approximately 20 per 1000 live births will require significant resuscitation at birth, and 10% of these infants will go on to have moderate-to-severe encephalopathy. Using currently available assessment methods, it is estimated that 20% of infants with significant hypoxic injury are clinically misclassified in the first hours of life and do not receive hypothermia (4). Thus, there is a critical need for improved biomarkers for prediction of grade of HIE and outcome (5).

Glial fibrillary acidic protein (GFAP), a monomeric intermediate filament protein predominant in astrocytes is known to display increased expression following acute brain injury or CNS degeneration (6). Previous studies, primarily carried out in adults have reported elevated levels of GFAP in traumatic brain injury, specifically related to focal mass lesions (7). Its use as a biomarker of HIE has previously been suggested, but the ability to differentiate between PA and HIE at birth has not been examined (1). Furthermore, little work has been carried out to determine the use of GFAP as a marker of moderate/severe HIE specifically to guide therapeutic intervention. To improve our ability to identify infants who will benefit from therapeutic hypothermia in time for effective intervention, any clinically useful biomarker will need to be reliably altered at or soon after birth.

Therefore, our aim was to examine the use of GFAP as an early clinical marker of HIE severity in full-term neonates by determining expression in umbilical cord blood (UCB) samples from healthy control infants, infants with perinatal asphyxia, and infants with HIE. We also wished to examine correlation of UCB GFAP with neurological outcome at 36 months.

## Materials and Methods

### Study Population

The BiHIVE Study (Biomarkers of HIE), including all recruitment, consenting, and sample processing procedures, was approved by the Clinical Research Ethics Committee of the Cork Teaching Hospitals. Through this study, any infant with suspected perinatal asphyxia or HIE in Cork University Maternity Hospital (CUMH), Ireland born between May 2009 and June 2011, was recruited as previously described (8). Briefly, infants >36 weeks gestational age were identified under strict enrollment criteria: cord pH <7.1 and/or Apgar score ≤6 at 5 min of life and/or requiring intubation or CPR at birth. Once infants were stable, parents were approached and informed about the study and written consent was obtained for each infant. All clinical and demographic information deemed relevant to the study was then recorded prospectively. Healthy matched controls were recruited through the BASELINE Study (www.baselinestudy.net), a longitudinal birth cohort study based in Cork. Controls were matched for sex, gestation, birth-weight, and gender, and all had uncomplicated deliveries.

### Cord Blood Sampling

Umbilical cord blood samples were collected immediately after delivery of all infants in this study and processed within 3 h following strict laboratory SOPs by a dedicated research team who were available 24 h a day. Samples were stored at −80°C in a monitored storage facility until analysis.

### Neonatal Assessment

All infants with suspected HIE received continuous multi-channel EEG monitoring. Our protocol for EEG monitoring has been previously described (9). Clinical grade of encephalopathy was assigned by a dedicated research fellow (BW) using the modified Sarnat score. Additionally, grade was further confirmed using EEG analysis with an experienced neonatal electroencephalographer (GB) who reviewed all EEG data. Therapeutic hypothermia was commenced at the discretion of clinicians blinded to the study data in all infants deemed to have moderate or severe encephalopathy using the TOBY registry treatment criteria and protocol (3).

### GFAP Analysis

Glial fibrillary acidic protein analysis was carried out by Banyan Biomarkers Inc., Alachua, FL, USA, on UCB serum samples using Banyan Biomarker’s proprietary sandwich Enzyme-Linked Immunosorbent Assays (ELISA) specific to GFAP. Detection levels for the Banyan Assay range from 0.03 to 50 ng/ml. All samples were run in duplicate and inter/intra assay variabilities of <10% were reported.

### Neurodevelopment Outcome

Where possible, developmental outcome was assessed at 36 months of age using the Bayley Scales of Infant and Toddler Development (Ed. III) (BSID-III) or the Ages and Stages Questionnaire (Ed. III) Ages and Stages Questionnaire (ASQ3) when the BSID-III was not possible due to reasons such as family relocation, etc. The Ages and Stages Questionnaire (ASQ3) is a parent-completed developmental screening questionnaire. It consists of 30 developmental items organized into five areas: Communication, Gross Motor, Fine Motor, Problem-Solving, and Personal–Social. Questionnaires for each child’s appropriate age interval were administered.

All BSID-III assessments were performed by a dedicated research fellow blinded to the clinical history of the patients (CA). For infants who underwent the assessment using the BSID-III, outcome was determined using the composite scores of the three subscales; cognitive, language, and motor. Infants were deemed to have an abnormal outcome if they scored ≤85 in two or more subscales or suffered neonatal death, cerebral palsy or autism. For infants who were assessed with ASQ3 only, results were considered abnormal if scores indicated the need for further assessment, falling >2 SD below the standardized mean, was advised in more than one area.

### Statistical Analysis

Statistical analysis was performed using IBM SPSS Statistics 22 (SPSS Inc., USA). Results were calculated using Student *t*-tests, Mann–Whitney, and Kruskal–Wallis tests, as appropriate.

## Results

### Study Population

In total, 169 infants were included in this study; 83 controls and 86 HIE cases. Of the 86 cases, 56 were classified as perinatal asphyxia without HIE, 21 with mild HIE, 5 with moderate HIE, and 4 with severe HIE, according to both the modified Sarnat assessment and EEG classification. Population demographics for the entire cohort are shown in Table 1.

**Table 1:** **Comparison of population demographics for entire study cohort (*n* = 169)**.

	Control	Perinatal asphyxia	HIE
	*n* = 83	*n* = 56	*n* = 30
Gestation (week + day)	40 + 3 (1 + 1)	40 + 3 (2 + 1)	40 + 3 (2 + 5)
Birth weight (g)	3518 (447.4)	3596 (533.8)	3495 (516.5)
Gender (M/F)	48/35	35/21	20/10
**Mode of delivery**
SVD	31 (37%)	18 (32%)	6 (20%)
Instrumental	36 (43%)	28 (50%)	15 (50%)
Elective cesarean section	4 (5%)	…	…
Emergency cesarean section	12 (15%)	10 (18%)	9 (30%)
1 min Apgar^a^	9 (9–9)	5 (3–7)	3 (1–5)
5 min Apgar^a^	10 (9–10)	8 (6–9)	5 (3–7)
Cord pH^a^	7.21 (7.15–7.26)	7.04 (6.99–7.09) ± 0.08	6.99 (6.91–7.08)

*Infants separated into controls, infants with perinatal asphyxia, and infants with hypoxic–ischemic encephalopathy (HIE). Data expressed as mean (SD) or median (interquartile rage) where appropriate*.

*^a^Represents a *p* value of <0.001 between groups calculated using Kruskal–Wallis or Mann–Whitney *U* tests*.

*M, male; F, female; SVD, spontaneous vaginal delivery*.

### GFAP Expression

**Figure 1:**
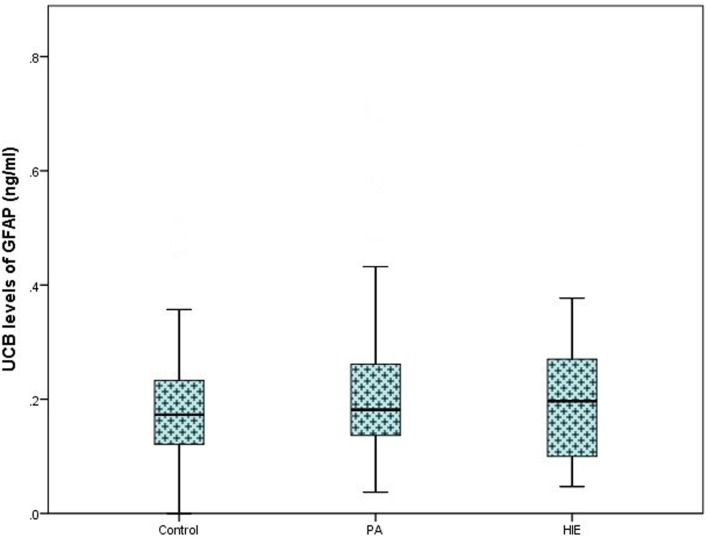
**Boxplot representing umbilical cord blood (UCB) levels of GFAP (ng/ml) following commercial ELISA analysis**. Infants grouped as healthy controls (*n* = 83), infants with perinatal asphyxia (PA) without HIE (*n* = 56), and infants with clinical and electrographically confirmed HIE (*n* = 30). No significant alteration was detected between groups (*p* = 0.566).

**Figure 2:**
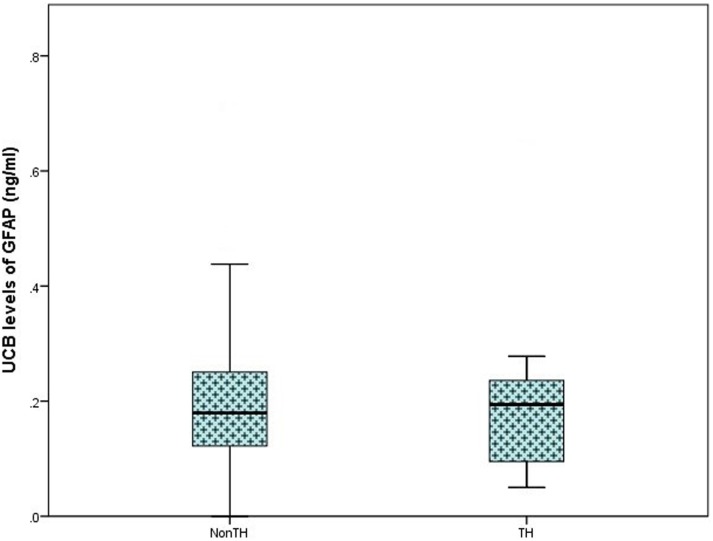
**Boxplot representing further analysis of umbilical cord blood (UCB) levels of GFAP (ng/ml) in infants which would be deemed eligible for therapeutic hypothermia (TH; moderate and severe hypoxic–ischemic encephalopathy, *n* = 9) and infants who would not meet the eligible criteria (Non-TH; controls, perinatal asphyxia, and mild hypoxic–ischemic encephalopathy, *n* = 160)**. No significant elevation in GFAP levels was observed (*p* = 0.919).

On analysis of ELISA results, there was no statistically significant difference in serum GFAP levels between case and control infants within this study (*p* = 0.287). Similarly, when case infants were grouped as PA without HIE (*n* = 56) and infants with HIE (*n* = 30), no difference was observed (*p* = 0.566, Figure 1). Additionally, when grade of HIE was specifically analyzed, no difference was observed between UCB levels of GFAP in mild, moderate or severe HIE (0.199 ± 0.095 ng/ml vs. 0.216 ± 0.087 ng/ml vs. 0.168 ± 0.258 ng/ml, *p* = 0.931). Finally, when grouped as infants who would be deemed eligible for therapeutic hypothermia (*n* = 9) vs. those who would not (*n* = 160), again no increase was observed (*p* = 0.919, Figure 2).

### Outcome at 36 Months of Age

**Figure 3:**
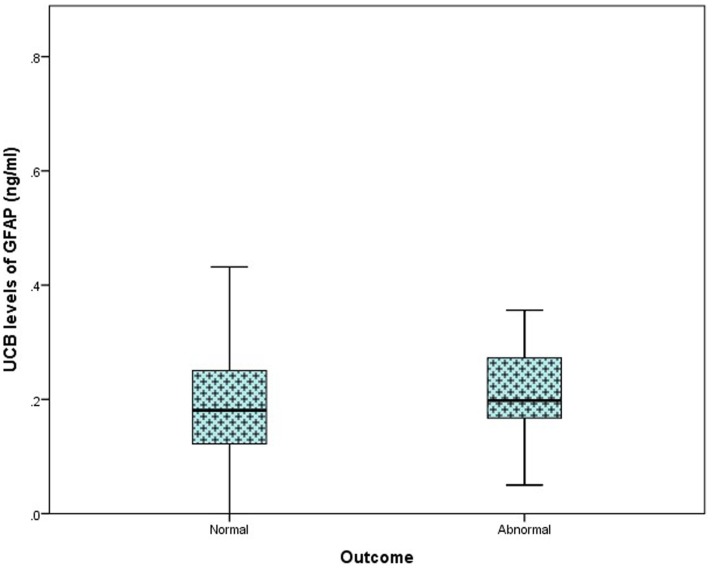
**Comparison of umbilical cord blood (UCB) levels of GFAP (nanogram per milliliter) from infants with a normal outcome at 24–36 months (*n* = 103) compared to those with an abnormal outcome (*n* = 13), presented as a box plot**. A significant difference was not detected between groups (*p* = 0.919).

In the total study population, outcome at 36 months was available in 116/169 (69%) infants [61 (73%) controls, 32 PA (57%), and 23 (77%) HIE]. Of these, 70 infants underwent outcome assessment using the Bayley Scales of Infant and Toddler Development (Ed. III). The remaining 46 infants underwent assessment using the Ages and Stages Questionnaire (Ed. III-ASQ3). Further breakdown of grade of HIE and outcome is available in Table 2. When classed as infants with a normal outcome vs. infants with an abnormal outcome at 36 months, no discernable elevation in UCB GFAP levels was observed Figure 3.

**Table 2:** **Comparison of neurodevelopmental follow-up at 36 months of age, primarily using the Bayley Scales of Infant and Toddler Development (Ed. III) (*n* = 70) or the Ages and Stages Questionnaire (Ed. III) (*n* = 46)**.

Outcome	Control (*n* = 61)	PA (*n* = 32)	Mild HIE (*n* = 15)	Mod/sev HIE (*n* = 8)
Normal	60	30	9	4
Abnormal	1	2	6	4^a^
GFAP (ng/ml)	0.20 (0.04–0.55)	0.22 (0.04–0.14)	0.20 (0.08–0.36)	0.23 (0.05–0.63)

*No alteration in GFAP levels was detected in the umbilical cord blood from healthy control infants, infants with perinatal asphyxia (PA), infants with mild HIE or infants with moderate or severe (Mod/Sev) HIE. GFAP levels (nanogram per milliliter) are expressed as mean (range)*.

*^a^Outcome defined by clinical diagnosis of CP or death*.

## Discussion

Increased levels of GFAP have been shown to be useful biomarkers of adult brain pathology (6, 10–15). In spite of this, investigations into its use in the neonatal field have been somewhat limited, with only a small number of publications suggesting GFAP as a biomarker in HIE with the ability to distinguish between grades of HIE (1, 2, 16–18). While these studies provided promising results, they also cited major limitations with regard to sample size and sample availability throughout. Post-natal blood samples were predominately used, with few studies determining levels in UCB, which is arguably the earliest possible source for a biomarker of HIE. However, as HIE is known to encompass a two stage evolving mechanism of injury, the time at which GFAP levels most represent degree of injury must be established.

In this study, our goal was to add to the current knowledge available for this promising biomarker specifically in relation to HIE. We aimed to validate the use of GFAP as a marker of HIE severity specifically and to overcome the previously cited limitations, mainly through the use of a larger, carefully defined population cohort and through the use of UCB as our sample source. All our samples are collected and processed in an operational biobank following strict SOPs. Additionally, UCB represents the earliest possible blood sample available after delivery. Although it is not taken directly from the infant, it provides a snapshot of an infant’s circulation at the time of delivery and can therefore offer a unique insight into the neonatal condition. This sample is relatively easy to obtain, does not involve an invasive procedure to an already stressed newborn and could potentially classify infants depending on degree of injury, contributing to rapid and reliable therapeutic decision-making.

A large collection of previously published research focused on GFAP and CNS damage in general is available; however, a recent review of neonatal biomarkers by Bersani et al. (19) called for further work to investigate the validity of this protein with a particular focus on outcome. We discovered no significant alterations in GFAP expression between our case and control groups or between different grades of HIE. Additionally, we found no correlation of GFAP levels with outcome at 36 months of age.

Our findings initially seem to contradict previously published research regarding the potential use of GFAP as an early biomarker of moderate/severe HIE. However, most studies to date have focused on post-natal samples, with significant elevation seen after 6–12 h (1, 18). More recently, a study focused on mixed cord samples in a historical cohort has shown no difference in serum GFAP levels between controls and infants with moderate-severe HIE (20). This is in keeping with previous work in adult TBI where peak levels occur at 12 h post-injury.

While there is good evidence that GFAP may be a useful marker of HIE severity in samples taken after six post-natal hours, we have not shown altered levels in UCB samples. This is the first prospective, carefully defined cohort study to focus on GFAP in UCB. This may limit the usefulness of GFAP as a biomarker to guide therapeutic intervention.

## Conclusion

Serum GFAP is not altered in the UCB samples of infants with perinatal asphyxia, with or without clinical and electrographic HIE, compared to normal controls. GFAP therefore may not be the ideal early biomarker to predict eligibility for therapeutic intervention.

## Conflict of Interest Statement

The authors declare that the research was conducted in the absence of any commercial or financial relationships that could be construed as a potential conflict of interest.
